# Supplementary material for: The interactome of intact mitochondria by cross-linking mass spectrometry provides evidence for coexisting respiratory supercomplexes
Source: Mol Cell Proteomics. 2017 Dec 8;17(2):216–32. doi: 10.1074/mcp.RA117.000470 (PMC5795388; doi:10.1074/mcp.RA117.000470)
Supplement: Supplemental Data [file supp_RA117.000470_133922_0_supp_23978_fzffwf.zip › spectra_annotation/mito_spectra_annotation/211-1-10-1-18-1.pdf]

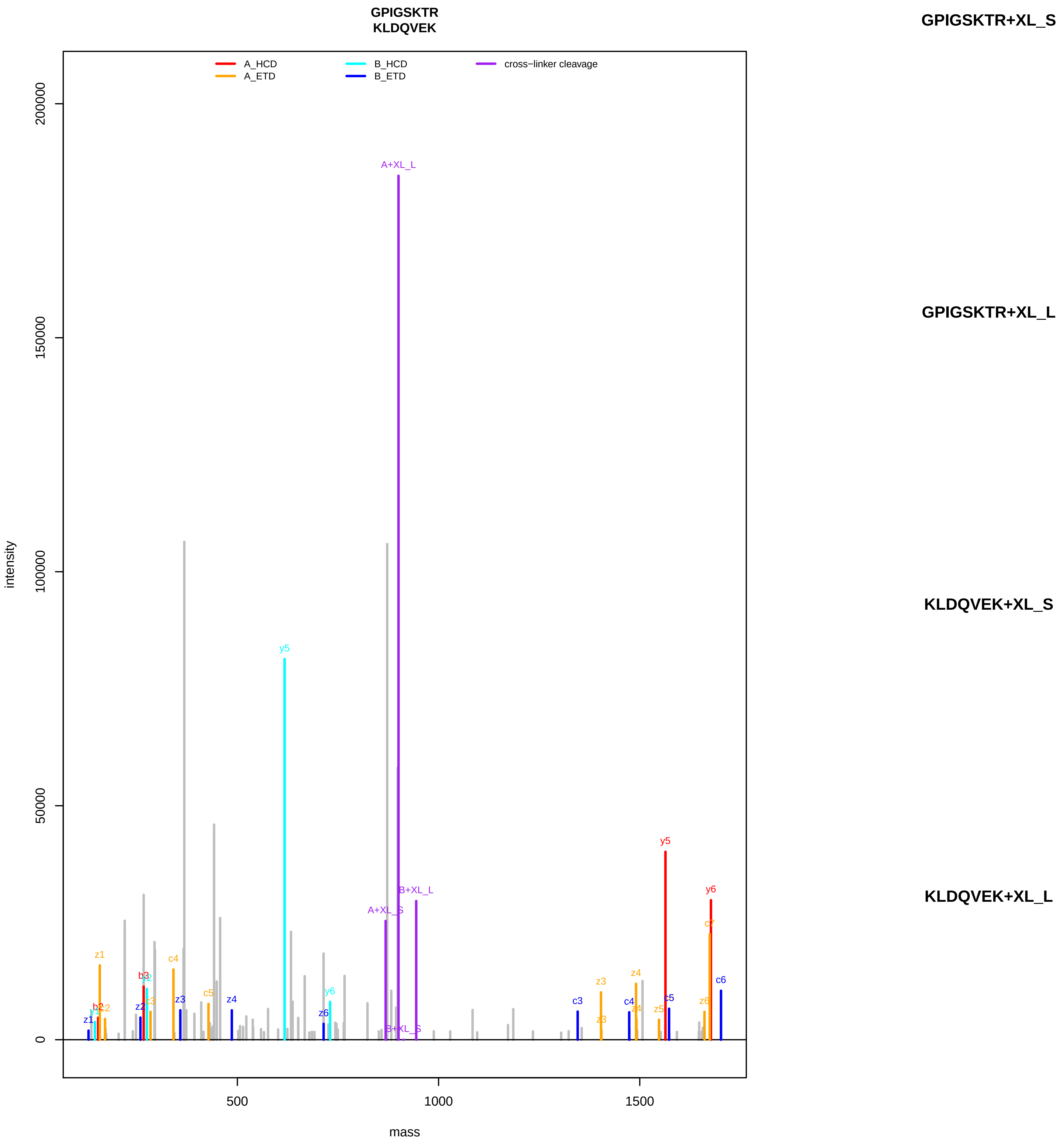
GPIGSKTR
KLDQVEK
A_HCD
A_ETD
B_HCD
B_ETD
cross−linker cleavage
A+XL_L
A+XL_S
B+XL_L
B+XL_S
y5
y6
c7
c6
c5
c4
c3
z1
z2
z3
z4
z5
z6
b2
b3
intensity
mass
0
50000
100000
150000
200000
500
1000
1500
GPIGSKTR+XL_S
GPIGSKTR+XL_L
KLDQVEK+XL_S
KLDQVEK+XL_L
